# Prevalence of Prosthetic Joint Infections in Patients With Haemophilic Arthropathy Treated at a Tertiary Hospital: A Retrospective Case-Control Study

**DOI:** 10.7759/cureus.110110

**Published:** 2026-06-02

**Authors:** Ntshuxeko Malepfana, Maxwell Jingo, Nkhodiseni Sikhauli, Johnny Mahlangu, Marule Paul Kgagudi

**Affiliations:** 1 Orthopaedic Surgery, University of the Witwatersrand, Johannesburg, Johannesburg, ZAF; 2 Haematology and School of Pathology, Faculty of Health Sciences, University of the Witwatersrand, Johannesburg, Johannesburg, ZAF; 3 Haematology and School of Pathology, Faculty of Health Sciences, National Health Laboratory Service (NHLS), Johannesburg, ZAF; 4 Haematology and School of Pathology, Faculty of Health Sciences, Charlotte Maxeke Johannesburg Academic Hospital, Johannesburg, ZAF

**Keywords:** haemophilia, haemophilic arthropathy, hiv, prosthetic joint infection, total joint arthroplasty

## Abstract

Background

Haemophilia is an X-linked inherited bleeding disorder that predisposes patients to haemophilic arthropathy (HPA), which can lead to the need for total joint arthroplasty (TJA). One of the major complications associated with TJA in this cohort is prosthetic joint infection (PJI). This study evaluated the prevalence, clinical characteristics, and microbiological profiles of PJI in haemophilic patients undergoing TJA at the Charlotte Maxeke Johannesburg Academic Hospital, Johannesburg, South Africa.

Methods

A retrospective comparative case-control study was performed, reviewing 208 patients (105 with HPA and 103 non-HPA controls) who underwent TJA between January 1, 2003, and December 31, 2023. Clinical, demographic, and laboratory data were collected, and PJI prevalence, common pathogens, infection-free survival, and the influence of human immunodeficiency virus (HIV) were analysed.

Results

The overall PJI rate was 4% in both the HPA and non-HPA groups. *Staphylococcus aureus* and *Klebsiella pneumoniae* were the most common pathogens. There was no significant difference in infection rates between the two groups. HIV status did not significantly affect the rate of PJI, and haemophilic patients had a longer infection-free survival.

Conclusions

Haemophilic patients undergoing TJA do not show a higher incidence of PJI compared to non-haemophilic patients. Effective clotting factor replacement therapy and infection control measures likely contribute to the reduced infection risk and longer infection-free survival.

## Introduction

Haemophilia is a rare X-linked inherited bleeding disorder that affects males [1]. The clinical presentation is usually that of spontaneous bleeding into soft tissues, with intra-articular bleeds being the hallmark of the disease at all ages [2,3]. The most frequently encountered types of haemophilia are A and B from mutations in factor VIII and factor IX genes, respectively [4-6], with a worldwide prevalence of 1/5000 for haemophilia A and 1/30,000 for haemophilia B [7,8]. Up to 90% of haemophilic patients experience episodic bleeds, 80% of which involve the knee, elbow, and ankle joints [9-11]. The most notable and disabling orthopaedic morbidity from haemophilia is the development of haemophilic arthropathy (HA), complicating recurrent intra-articular bleeds with a joint predilection [3,6,7]. Clinically, an affected joint progresses into persistent pain, restriction of joint motion, and functional limitation leading to disability [3,7].

Aggressive treatment of haemophilia with factor replacement can prevent recurrent bleeding and, therefore, HA [12,13]. Several haemophilia treatment options have been described in the literature, and adherence can help reduce the need for total joint arthroplasty (TJA), which is associated with complications such as prosthetic joint infection (PJI) [14-17]. PJI is reported in 0.5% to 2.6% of primary hip and knee arthroplasties and up to 25% of cases of revision arthroplasties. Histopathology is considered the gold standard of diagnosis for PJI, since laboratory studies can be poorly sensitive and specific [18,19]. Different strategies for PJI prevention have been described in the past; however, it still develops, especially in high-risk patients [20-22].

Despite the extensive research on HA and TJA, to date, there are no studies that have looked at the “prevalence of prosthetic joint infection in haemophilia patients treated with joint arthroplasty.” Anecdotal evidence shows that our patients wait longer for both primary and revision arthroplasties. In addition, South Africa potentially has a unique population of patients living with both haemophilia and human immunodeficiency virus (HIV). Therefore, there is a need to conduct a study to analyse PJI in haemophilic patients treated with joint arthroplasty in our context. To our knowledge, no study has been published on PJI in HA treated with TJA.

This article was previously presented as a meeting abstract at the 71st South African Orthopaedic Association Congress on September 4, 2025, the University of the Witwatersrand School of Clinical Medicine Biennial Research Day on September 18, 2025, the University of the Witwatersrand Orthopaedics Department Research Day on November 5, 2025, and the South African Haemophilia Foundation MASAC Educational Symposium on November 7, 2025. 

## Materials and methods

This was a retrospective, comparative case-control study conducted at the Charlotte Maxeke Johannesburg Academic Hospital (CMJAH), in Johannesburg, South Africa. The study investigated the prevalence and clinical characteristics of PJIs in haemophilic patients who underwent TJA from January 2003 to December 2023, allowing for the inclusion of a substantial cohort of both haemophilic and non-haemophilic patients undergoing TJA. The study was approved by the Human Research Ethics Committee (HREC) of the University of the Witwatersrand (Clearance No: M240858) and the hospital’s research ethics committee. Written informed consent was waived due to the retrospective nature of the study.

The study population included HA patients and non-HA patients. The HA patients (haemophilic group) consisted of patients diagnosed with haemophilia A or B with a documented history of recurrent joint bleeds and diagnosed HA, who underwent TJA as part of their management of HA. The non-HA patients (control group) were patients with no diagnosis of haemophilia who had TJA for various non-haemophilic reasons, such as osteoarthritis, rheumatoid arthritis, or trauma-related joint damage. The non-haemophilic group was the control to assess whether haemophilic patients had a higher incidence of PJI.

The inclusion criteria for both groups were (1) aged 18 years and older at the time of surgery, (2) patients who had undergone primary TKA or THA during the study period, and (3) haemophilic patients with a confirmed diagnosis of haemophilia A (factor VIII deficiency) or haemophilia B (factor IX deficiency), documented in their medical records. The control group consisted of patients who had undergone primary joint arthroplasty for reasons unrelated to haemophilia (e.g., osteoarthritis, trauma). Both groups were required to have complete medical records, including preoperative data, surgical outcomes, and postoperative follow-up information.

Patients were excluded if (1) they had missing key clinical data such as laboratory results, detailed surgical records, or infection status; (2) they had undergone revision surgeries or had complications from previous joint replacements, to focus on primary joint arthroplasties; and (3) they had pre-existing joint infection or other major surgical contraindications.

Data collection was retrospective, sourced from CMJAH's patient health records. Key data points included demographic information (age, gender, race, BMI), clinical characteristics (haemophilia type, comorbidities, HIV status), surgical data (joint replacement type - total knee arthroplasty (TKA) vs total hip arthroplasty (THA); intraoperative blood loss; side of surgery (left vs right); clotting factor replacement before, during, and after surgery), infection data (PJI diagnosis, microbial pathogens, antibiotic resistance patterns and sensitivity, antibiotic therapy, and postoperative management), and follow-up data (duration, infection-free survival).

Descriptive and inferential statistical methods were used to analyse the data. Statistical analysis was conducted using Stata version 18 (StataCorp, College Station, TX, USA).

The following steps were undertaken: (i) Descriptive Statistics: Categorical variables (e.g., infection status, gender, race, presence of comorbidities) were summarized using frequencies and percentages. Continuous variables (e.g., age, BMI, blood loss, CD4 count) were summarized using means and standard deviations (for normally distributed data) or medians and interquartile ranges (for skewed data). (ii) Comparative Analysis: The chi-square test or Fisher’s exact test was used to compare categorical variables (e.g., infection rates, microbial pathogens) between the HPA and non-HPA groups. Independent t-tests (for normally distributed data) or Mann-Whitney U tests (for non-normally distributed data) were used to compare continuous variables (e.g., age, BMI, blood loss) between the two groups. Kaplan-Meier survival analysis was employed to estimate infection-free survival rates in the two groups. The log-rank test was used to compare survival distributions between HPA and non-HPA patients. (iii) Multivariate Analysis: Univariate analysis was first performed to identify potential risk factors for PJI. Variables with a p-value < 0.1 in univariate analysis were included in multivariate logistic regression analysis to identify factors independently associated with PJI. This included variables such as age, joint type, haemophilia status, blood loss, and HIV status. A p-value < 0.05 was considered statistically significant for all analyses. (iv) Antibiotic Resistance Patterns: The antibiotic resistance and sensitivity patterns for pathogens isolated from PJI cases were analyzed using standard microbiological methods. Resistance rates to common antibiotics, such as amoxicillin, meropenem, and cloxacillin, were documented and compared between the two groups. (v) Sensitivity Analysis: To assess the robustness of the findings, sensitivity analyses were performed based on different categories of HIV status (positive vs negative) and stratified by joint type (TKA vs THA).

## Results

**Figure 1:**
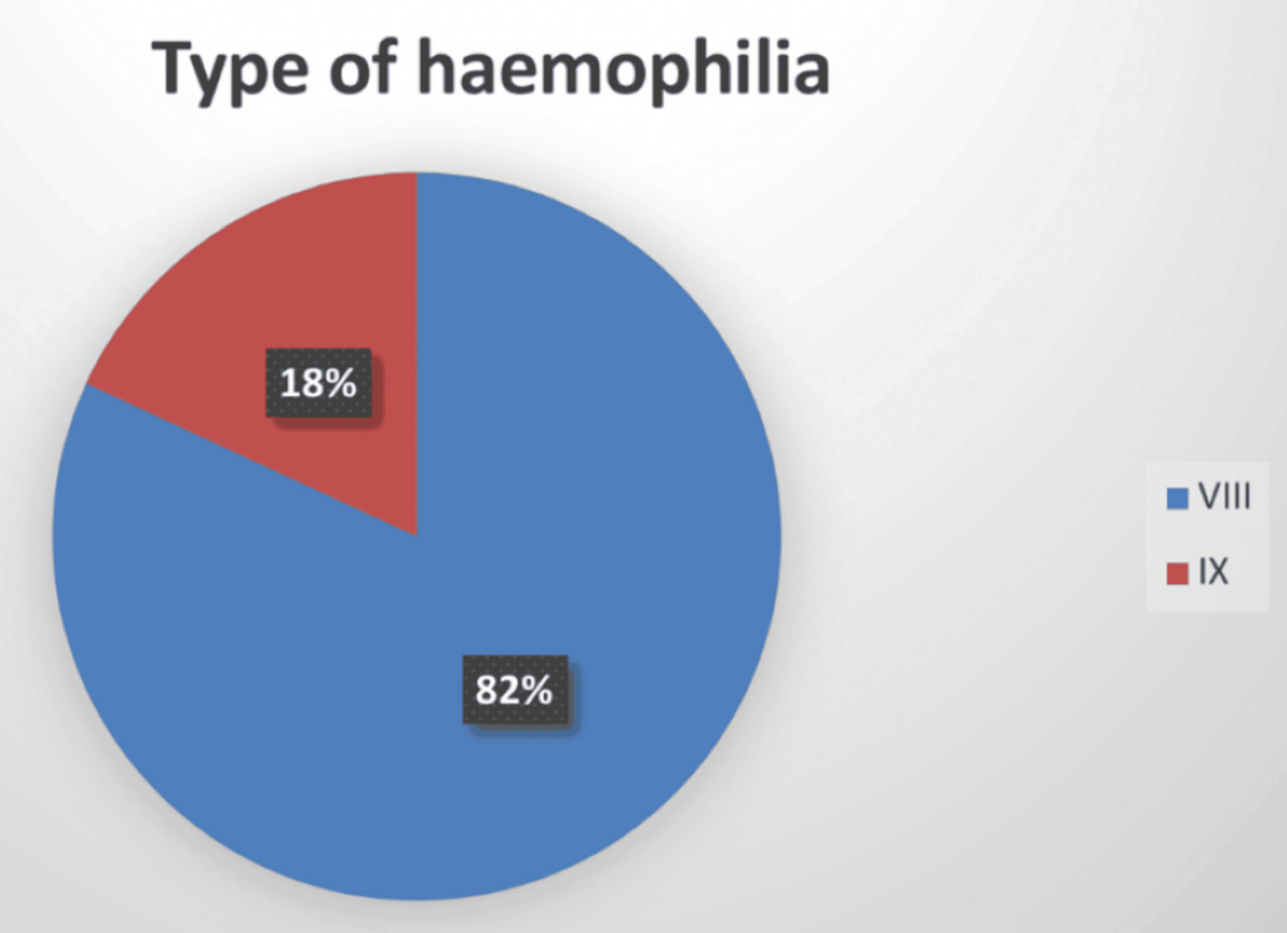
Pie chart demonstrating the distribution of haemophilia types in the study cohort (N = 105)

**Figure 2:**
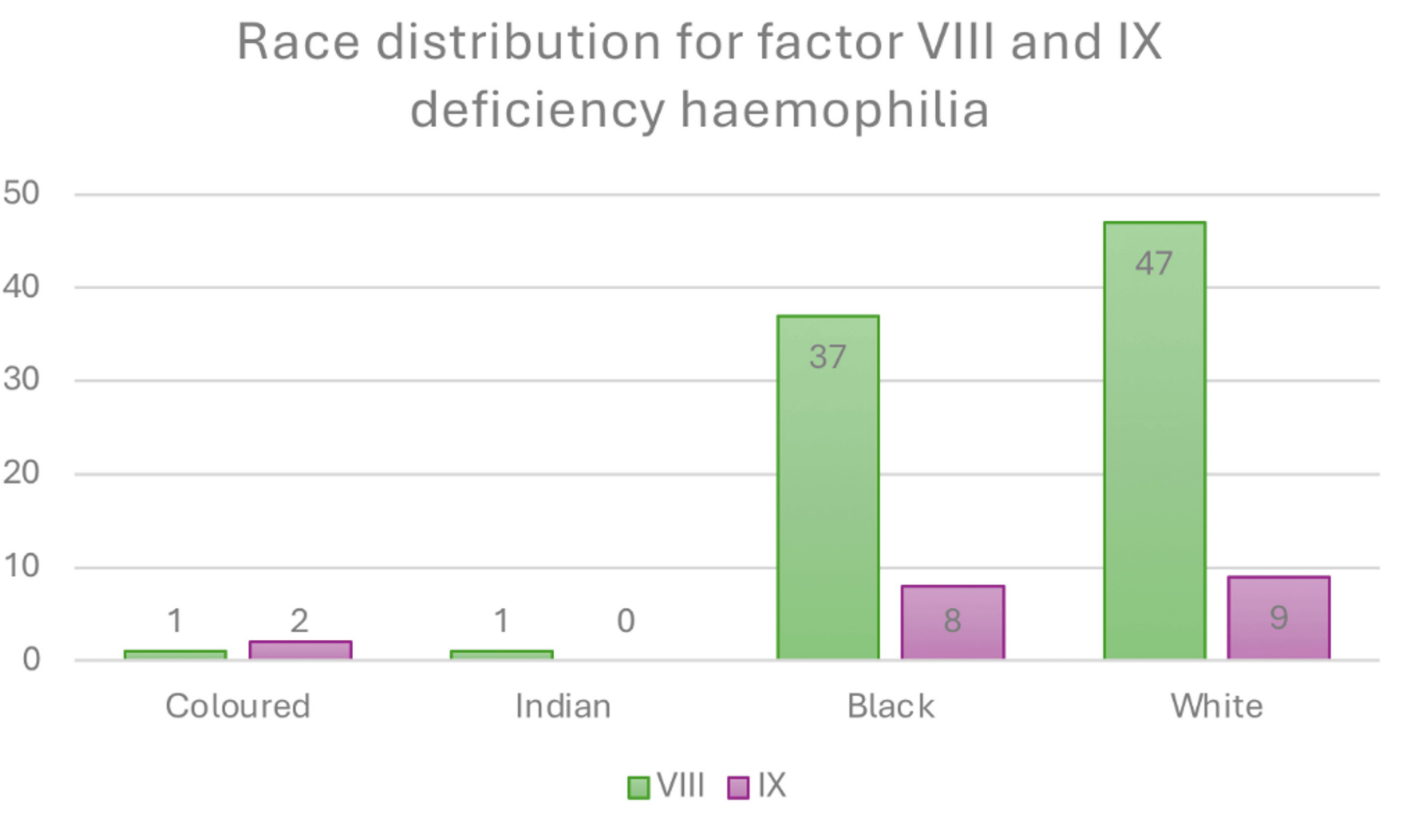
Race distribution

A total of 208 patients met the inclusion criteria; 105 had HA (79 TKRs and 26 THRs), and 103 were non-HA controls. The haemophilic patients were matched with non-haemophilic males. The median age for all patients was 55 years. The HA patients were notably younger (median 48 (46-51) vs. 62 (60-64) years, p < 0.00001). White patients were affected slightly more (53%) than Black patients (45%). Factor VIII deficiency was the most common type of deficiency, affecting 86/105 (82%) individuals (Figure 1). Figure 2 shows haemophilia factor deficiency type by race. Table 1 presents a summary of the study results, comparing the haemophilic and non-haemophilic groups.

**Table 1:** Summary of the study results showing comparison between haemophilic and non-haemophilic group BPH: benign prostatic hyperplasia; HPT: hypertension

	Total	Case	Control	p-value
Age	55 (48-62)	48 (46-51)	62 (60-64)	<0.00001
HIV status				
Negative	201 (97)	101 (96)	100 (97)	1.000
Positive	7 (3)	4 (4)	3 (3)	
CD4 cell count	650 (375-750)	725 (675-758)	375 (374-642)	0.0339
Viral load	LTDL	LTDL	LTDL- <20	1.000
Follow-up period	9 (6-12)	12 (5-16)	6 (5-7)	<0.00001
Infection-free period	6 (4-8)	8 (4-12)	4 (1.5-8)	0.2425
Organism				0.657
Acinetobacter baumannii	1 (14)	0	1 (25)	
Klebsiella	3 (43)	1 (33)	2 (50)	
Staphylococcus aureus	3 (43)	2 (67)	1 (25)	
Resistance				0.600
Amoxicillin/ampicillin	3 (42.86)	1 (33)	0	
Amoxicillin/penicillin	2 (28.57)	0	2 (50)	
Meropenem	1 (14.29)	0	1 (25)	
Penicillin/ampicillin	1 (14.29)	0	1 (25)	
Sensitivity				0.657
Amikacin	1 (14.29)	1 (33.33)	0	
Cloxacillin	3 (42.86)	2 (66.67)	1 (25)	
Augmentin	1 (14.29)	0	1 (25)	
Colistin	1 (14.29)	0	1 (25)	
Ertapenem	1 (14.29)	0	1 (25)	
Comorbidities				-
BPH	4 (44)	0	4 (44)	
HPT	4 (44)	0	4 (44)	
Hyperthyroidism	1 (12)	0	1 (12)	
Sepsis				1.000
No	200 (96.15)	101 (96.19)	99 (96.12)	
Yes	8 (3.85)	4 (3.85)	4 (3.88)	

**Figure 3:**
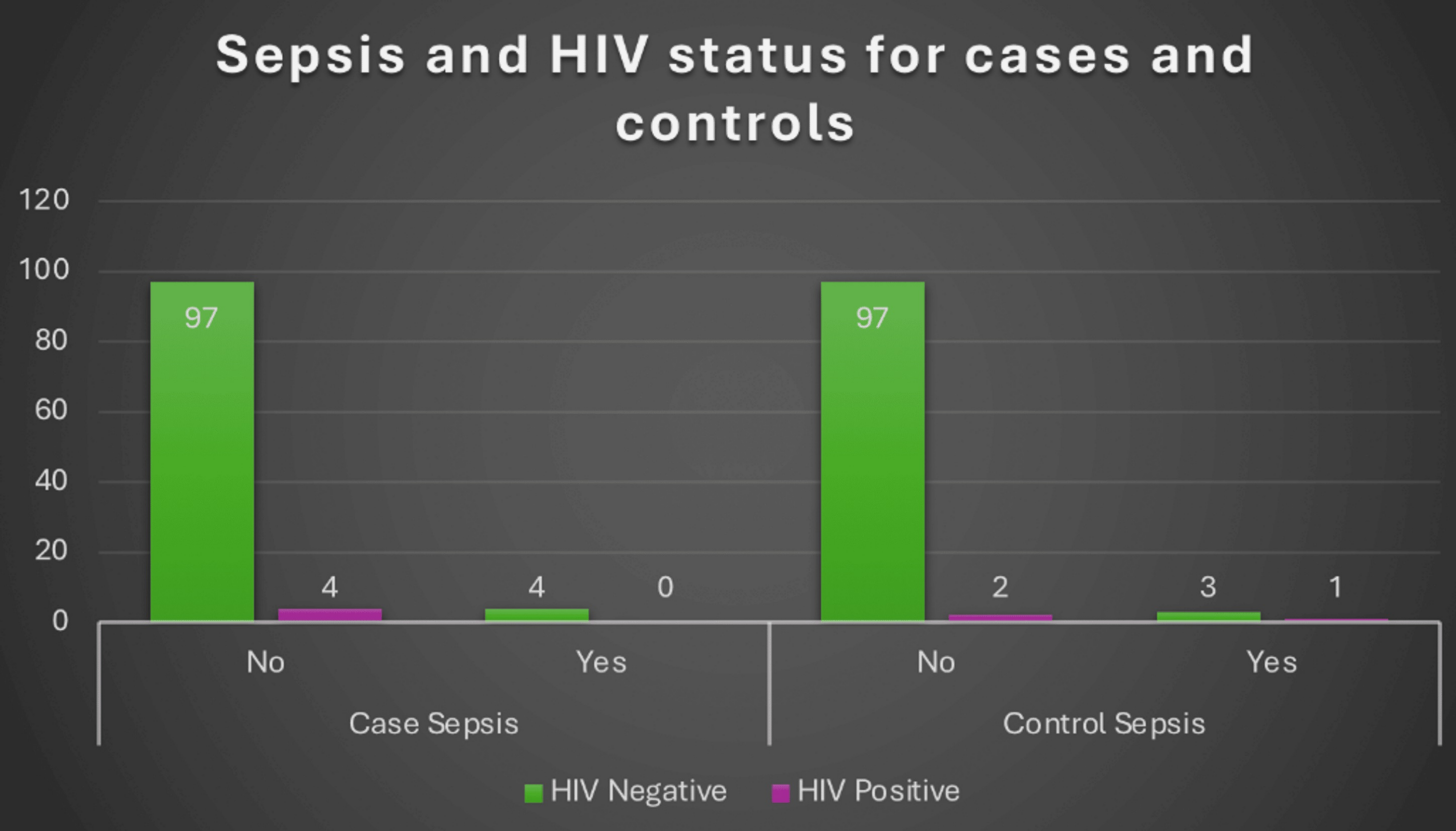
PJI rate and HIV status: graphical illustration PJI: prosthetic joint infection

**Figure 4:**
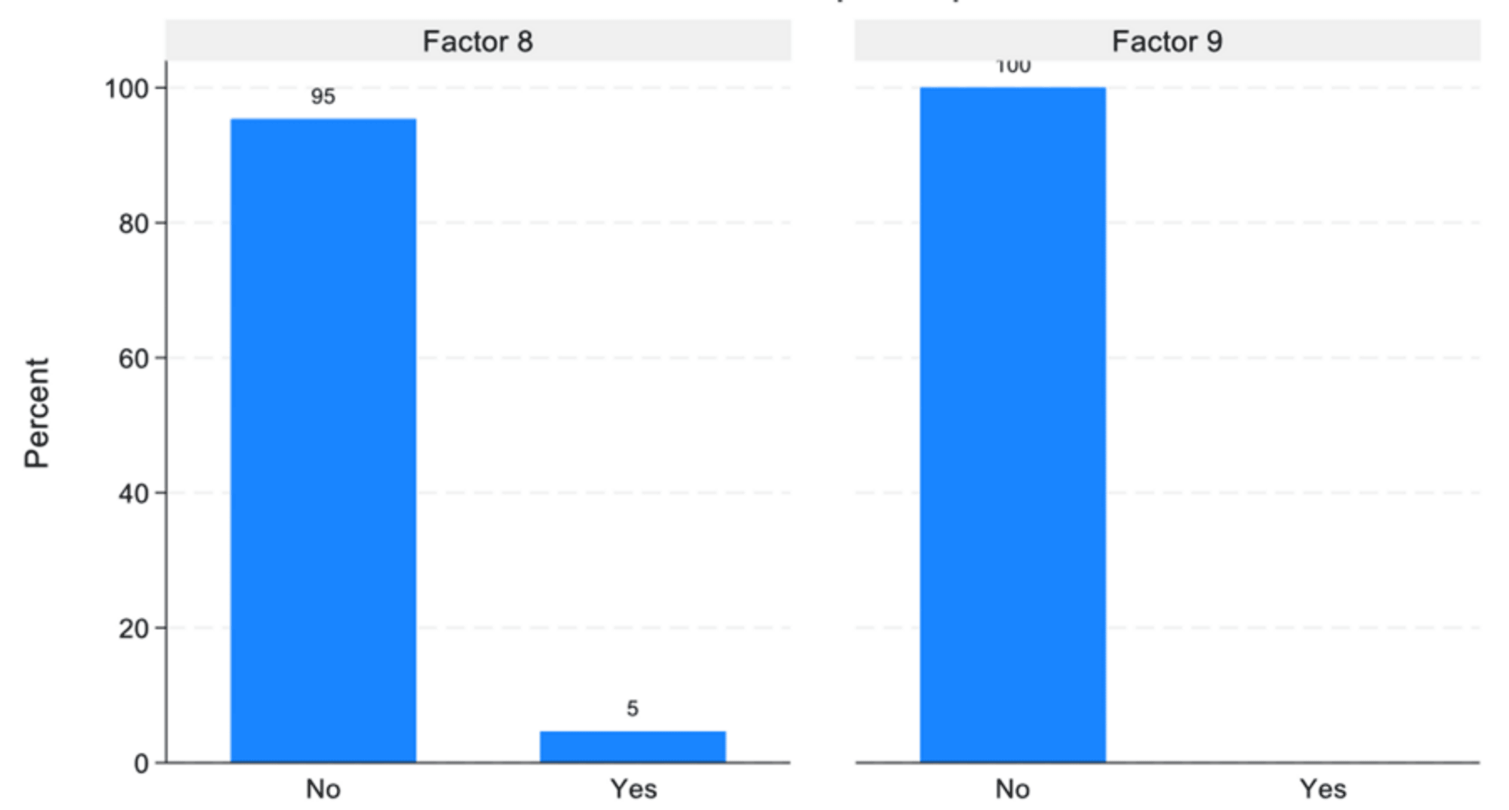
Infection rate in haemophilic patients

Seven patients were HIV-positive (four HA and three non-HA), with statistically significant viral suppression in the HA group (below detectable levels) and higher CD4 counts (725 vs 375, p = 0.0339) (Figure 3). Figure 4 shows PJI based on HIV status. 

**Figure 5:**
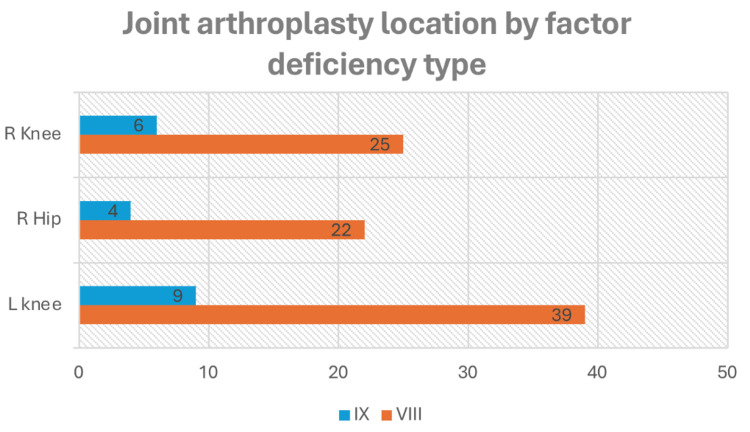
Arthroplasty site

PJI was seen only in the factor VIII deficiency group. *Klebsiella pneumoniae* and *Staphylococcus aureus* were the most common pathogens (43% each). While *S. aureus* was more frequent in the HA group (67% vs 25%), statistical significance was not reached (p = 0.657). The PJI rate was equal in both groups (4%). Figure 5 depicts the arthroplasties done by site and side of the body.

Antibiotic resistance was concerning, with 42.86% resistance to amoxicillin-ampicillin and 14.29% to meropenem, highlighting challenges in empiric treatment. Sensitivity testing revealed cloxacillin as the most effective agent (42.86%) for *S. aureus*, although resistance to other first-line drugs was prevalent (Table 1).

**Figure 6:**
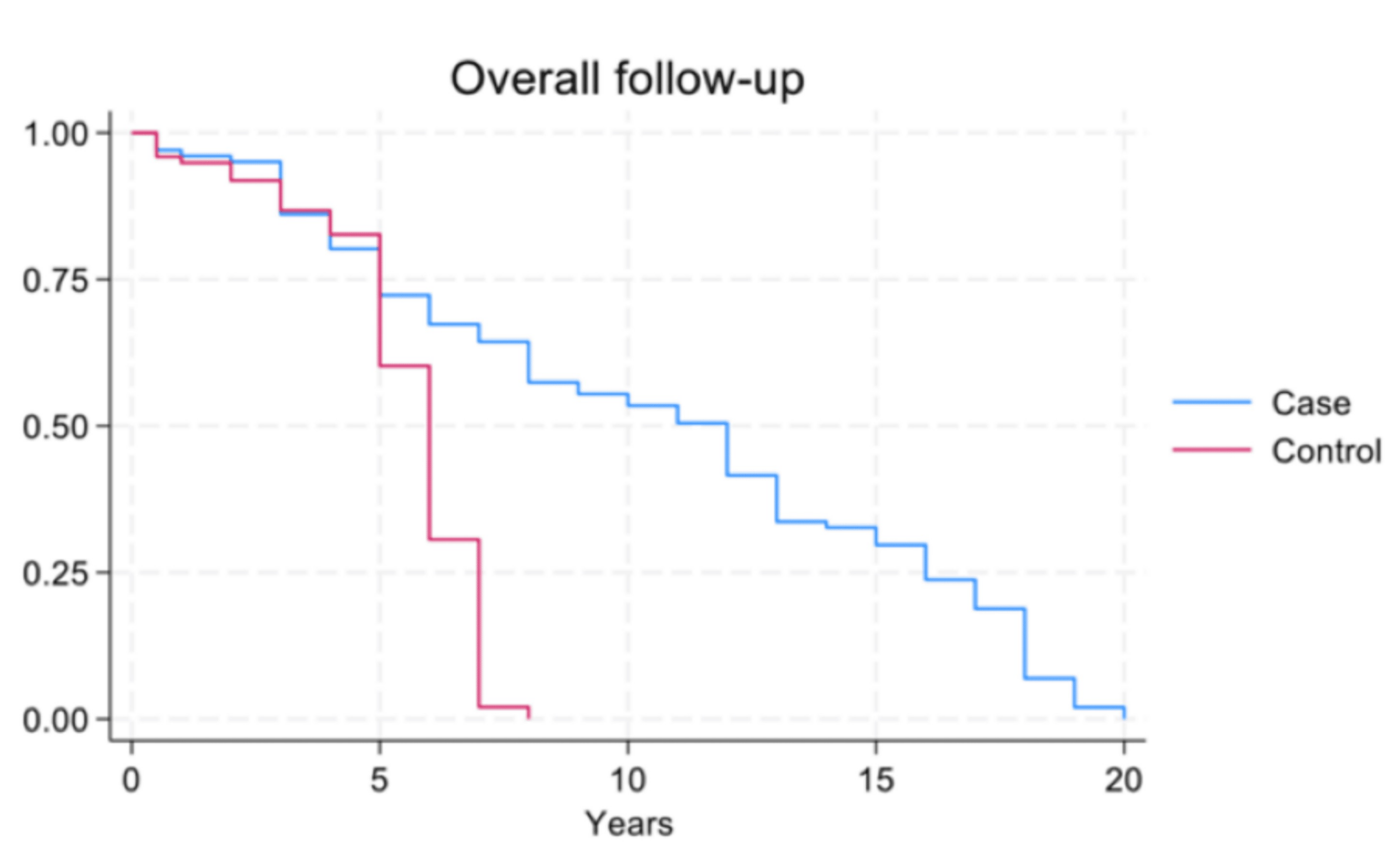
Overall follow-up for both cases and controls

**Figure 7:**
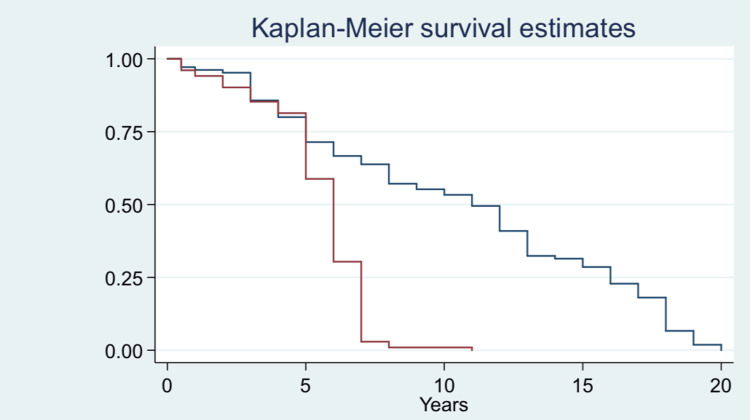
Median infection-free years

The median follow-up was 12 years (5-16, IQR) and 6 years (5-7, IQR) for the HA and non-HA groups, respectively. The median overall follow-up was 11 years (5-16) for factor VIII deficiency and 12 years (7-16) for factor IX deficiency (Figure 6). However, infection-free survival did not differ significantly (8 vs 4 years, p = 0.2425) and was comparable in both groups (Figure 7).

## Discussion

In our study, we had 105 haemophilic patients, with 79 knees and 26 hips. Wang et al. had a sample size of 28 patients (32 knees), and Jiang et al. had 87 patients (117 knees) [23,24]. A retrospective study by Hosseini et al. identified 42 patients (46 TJA) [25], while Liu et al. reported 71 patients (78 knees) with HA [26]. Our study sample is comparable to other studies in the literature. The median age for overall patients was 55 years, while our cases were notably younger (median 48 vs. 62 years, p < 0.00001). Factor VIII deficiency was the most common deficiency; the majority of haemophilia A patients were White (Figure 3). Liu et al. reported a mean age at the time of surgery of 38.4 ± 7.9 years (range: 21-63 years), while Wang et al. reported an average age of 38.6 years [23,25]. Haemophilia A was the most common type in our study, and our age range was 46-51 years, consistent with other international studies [27-29]. In our study, there was a longer waiting period, which can be associated with a lack of resources [30-34].

Even though the literature suggests that haemophilic patients commonly have hypertension, our findings were contrary, with no cases of hypertension reported. This might be because we relied on medical records rather than measuring blood pressure, as well as having a younger study population. However, another study found haemophilia to be protective against cardiovascular disease, which could explain our hypertension findings [30,35]. Our prevalence of HIV was low (4%) in both groups in our study, with statistically significant viral suppression in the HA group and higher CD4 counts (725 vs. 375, p = 0.0339). Interestingly, there was no PJI in our HIV-positive haemophilic patients. According to Beckers et al., 17% of HIV-positive haemophilic patients developed PJI [31]. Currently, there is no consensus on whether HIV infection increases the rate of PJI in haemophilic patients [31]. However, our findings were consistent with those of Wang et al., who reported a low infection rate associated with HIV [23]. Challoumas et al. reported a higher incidence of PJI in TKA compared to THA [27]. Rodriguez-Merchan et al. also analysed 19 TKAs in haemophilic patients, with only one knee developing PJI in that study [17]. Rodriguez-Merchan et al. analysed 107 TKAs in 74 patients, and seven knees developed PJI [17]. In 2022, He et al. reported on the clinical outcomes and adverse event rates for bilateral THA in haemophilia A patients, with no PJI cases [7,13]. In our study, we reported a lower prevalence of PJI in HA (3.8%). This figure is comparable to that of the non-HA population globally [9,24]. Our results are in keeping with the most recent studies on TKA in patients with HA, which also report similarly low infection rates [9,23]. This may be due to a good clotting factor replacement programme, counselling, and strict infection control measures, as well as surgeries being performed by experienced surgeons.

*K. pneumoniae* and *S. aureus* were the most common pathogens isolated in our study, at 43% each. While *S. aureus* was more frequent in the HA group (67% vs. 25%), statistical significance was not reached (p = 0.657). Most studies have reported *S. aureus* and coagulase-negative staphylococci as the common organisms responsible for PJI [35]. Challoumas et al. reported *S. aureus* (58%) as the most common causative organism [27]. In our setting, *K. pneumoniae* was associated with an outbreak around the study period, affecting the non-HA group of patients. Our finding was consistent with the literature, with *S. aureus* being the most common organism in the HA group [33]. Antibiotic resistance was concerning, with 42.86% resistance to amoxicillin/ampicillin and 14.29% to meropenem, highlighting challenges in empiric treatment. Sensitivity testing showed cloxacillin as the most effective agent against *S. aureus* (42.86%), though resistance to other first-line drugs was prevalent. Fortunately, we did not observe any methicillin-resistant *S. aureus* (MRSA) isolates, as reported in other studies [34].

Rodriguez et al. analysed 19 TKAs in haemophilic patients in 1989, with a 9.5-year follow-up, and one patient developed PJI [17]. Rodriguez-Merchan et al., in a follow-up study, analysed 107 TKAs in 74 patients with HA, focusing on prosthetic survival and PJI. With a follow-up of 11 years, only seven knees developed PJI [17]. Wang et al., with a mean follow-up of 69.1 months, also had no infections to report [23]. Our study had a median follow-up of 12 years and 6 years for HA and non-HA controls, respectively, with comparable infection-free survival in both groups. According to Mortazavi et al., the rate of PJI, especially in haemophilic patients, is not directly proportional to the duration of follow-up, with infections being reported at extremes of follow-up [35]. HIV infection, frequent intravenous infusion of coagulation factors, and a high incidence of haematoma are all reported risk factors for PJI in a population prone to haematoma formation [35]. Overall, blood loss was minimal in our cohort, which can be attributed to good peri-operative haemostasis and intraoperative soft tissue care by surgeons. Interestingly, Shen et al. reported a greater risk of blood loss in the elderly haemophilic population [36], while Rodriguez-Merchan et al. reported the mean blood loss volume during TKA for HA of the knee to be 542.3 mL [37]. Carulli et al. reported a mean blood loss of 120 mL and 610 mL for TKA and THA, respectively [32]. This may highlight variations in treatment protocols for HA patients across different sites. Two revision surgeries are typically reported in patients who develop PJI, with patients requiring a second revision for better outcomes; however, in our series, none required revision arthroplasty [17].

## Conclusions

To our knowledge, this is the first study globally to compare the prevalence of PJI in haemophilic and non-haemophilic patients. It is still not clear whether haemophilic patients are at an increased risk of PJI compared to the general population. We hypothesized that haemophilic patients are at an increased risk of infection. This study demonstrates that haemophilic patients do not exhibit a higher risk of PJI compared to non-haemophilic patients undergoing TJA. The findings highlight the importance of comprehensive perioperative care in minimizing infection risk and suggest that well-managed haemophilic patients can undergo joint replacement surgery safely. Further prospective, multicentre studies are necessary to confirm these findings and explore additional factors that may influence infection risk and long-term outcomes in this population.

Several limitations of this study must be acknowledged. The retrospective design is a major limitation, as it depends on the accuracy and completeness of medical records, which can introduce bias, especially in the identification and reporting of infections. Additionally, reliance on clinical documentation without prospective infection surveillance may have resulted in an underestimation of infection rates. Furthermore, the study was conducted at a single institution, limiting the generalisability of its findings. Variations in surgical techniques, infection control protocols, and patient management practices at different centres could influence PJI rates and may not be fully reflected in the study results. Although the sample size of 208 patients is relatively large, subgroup analyses, such as comparing HIV-positive and HIV-negative haemophilic patients, are limited. The small number of infections in the haemophilic group (four infections) also reduces the statistical power of some comparisons. The diagnosis of PJI was based on clinical presentation and microbiological cultures, and the absence of advanced imaging techniques or joint aspiration in all cases may have led to missed or delayed diagnoses of infection.
